# *Lycium barbarum* Berries (Solanaceae) as Source of Bioactive Compounds for Healthy Purposes: A Review

**DOI:** 10.3390/ijms24054777

**Published:** 2023-03-01

**Authors:** Filipa Teixeira, Ana Margarida Silva, Cristina Delerue-Matos, Francisca Rodrigues

**Affiliations:** REQUIMTE/LAQV, ISEP, Polytechnic of Porto, Rua Dr. António Bernardino de Almeida, 4249-015 Porto, Portugal

**Keywords:** goji berries, pro-healthy effects, phenolics, biological properties, functional ingredients

## Abstract

*Lycium barbarum* L. is a species widely used in dietary supplements and natural healthcare products. The berries, also known as goji or wolfberries, mostly grow in China, but recent reports on their outstanding bioactive properties have increased their popularity and cultivation around the world. Goji berries are a remarkable source of phenolic compounds (such as phenolic acids and flavonoids), carotenoids, organic acids, carbohydrates (fructose and glucose), and vitamins (ascorbic acid). Several biological activities, such as antioxidant, antimicrobial, anti-inflammatory, prebiotic, and anticancer activities, have been associated with its consumption. Hence, goji berries were highlighted as an excellent source of functional ingredients with promising applications in food and nutraceutical fields. This review aims to summarize the phytochemical composition and biological activities, along with various industrial applications, of *L. barbarum* berries. Simultaneously, the valorization of goji berries by-products, with its associated economic advantages, will be emphasized and explored.

## 1. Introduction

Plants have been used for thousands of years as a source of compounds for traditional medicine, aiming to prevent and treat health problems [1]. Due to a broad number of studies that describe the influence of natural products on the human endogenous defense system [2,3] as well as “curative” effects against a wide spectrum of disorders, including cardiovascular and neurodegenerative diseases, obesity, and certain types of cancer [4], multiple natural products have been used for healthcare proposes. Nowadays, the interest in exotic berry-type fruits has expanded worldwide [5,6,7,8]. Society has become more concerned with eating habits, mostly due to the reinforcement of a positive relationship between good eating habits and the prevention of disease development, particularly diabetes and cardiovascular and neurological pathologies [9]. Simultaneously, the emergent awareness of the planet and the impact that various types of industries have on it has boosted the population’s concerns and demands for greener formulations with bioactive ingredients recovered from natural sources [5,10]. Therefore, the consumption of natural matrices has increased in recent years, not only as supplements for imbalanced diets but also as an integral part of a normal healthy diet [7,11].

Recently, several reports highlighted the impressive bioactive capacities of goji berries [2,4,10,12,13,14,15,16], not only in cell assays [17,18,19] but also in animal studies [3,14,20] and human trials [21]. Despite the growing numbers of published papers regarding the bioactive composition of goji berries, few of them give particular emphasis to in vivo studies as well as to the by-products generated during berry production. Therefore, the aim of this work is to provide a comprehensive review of the bioactive compounds of goji berries, along with their biological activity, giving a particular focus to in vivo studies and clinical trials. The various industrial applications of *L. barbarum* berries will also be highlighted, as well as the valorization of goji berries by-products.

## 2. *Lycium barbarum* L.

*Lycium barbarum* L. is one of the most common species of the Solanaceae family [13,22]. The berries traditionally grow in China, Tibet, and other parts of Asia [6,13,23]. China is the primary worldwide supplier [24,25], with about 25,000–30,000 tons of dried fruit annually produced in Ningxia, Xinjiang, Gansu, Qinghai, and Mongolia [26]. Asia is the region with the highest production (71.2%), followed by Africa (15.8%), America (8.3%), Oceania (3.4%) and Europe (1.3%) [27]. Due to the rising reports of the positive correlation between the consumption of natural matrices and health improvement [9], the production of fruit has been increasing in the last 20 years all over the world. This is the case of goji berries, whose production has been increasing in the last decades [28], particularly in Europe (Italy, Romania, Bulgaria, Portugal, Greece, Serbia), Northern America, and Australia. Currently, Romania has the largest cultivated area of *L. barbarum* in the European Union [4,10]

**Figure 1:**
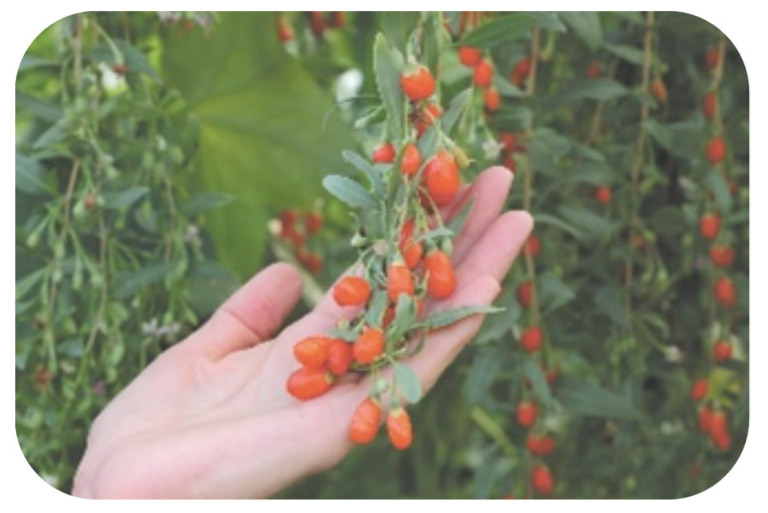
*L. barbarum* L. berries [29].

The goji berries can be divided into different classes according to their ripening stage, dimension, weight, color, firmness, solid soluble content, pH, and titratable acidity [22]. The mature fruit (Figure 1) is between 1 and 2 cm long, presenting an ellipsoid shape and a bright orange-red color, similar to a mature mini-tomato, and contains between 20 and 40 tiny seeds per fruit [6,13,22].

The berries have a sweet taste [16] and are widely used as a dietary supplement and natural health product [4,23,25]. Although mostly consumed fresh in the regions of cultivation [9], around the world, goji berries are essentially consumed dried [9,12] or transformed into alimentary products, such as juices, herbal teas, yogurt products, granola, powders, and tablets, among others [9,12,30]. The most commonly sold goji berry-based products are beverages, wine, juice, tea, and concentrates [4,14,23]. A popular goji berry juice brand, “GoChi”, has shown increasing effectiveness as an antioxidant, giving rise to subjective feelings of general well-being as well as improvements in neurologic/psychologic performance and human gastrointestinal functions [23], which has led to huge popularity among consumers.

For thousands of years, goji berries have been used as herbal medicine in Asian countries [24]. Based on their rich nutritional value and medical properties, such as their antioxidant, antimicrobial, immunomodulatory, and anti-inflammatory effects [15,23,31], the fruit has been employed as an anti-aging treatment, tranquilizer, and thirst-quenching treatment [2,13]. As a folk medicine, *L. barbarum* fruits have been employed by the local population for blood nourishing, in the treatment of early onset diabetes, tuberculosis, dizziness, and chronic cough, and for the protection of eye health [15].

All these pro-healthy properties have attracted the attention of consumers to goji juices and fruits, transforming goji berries into one of the most popular functional food ingredients/supplements worldwide [13]. Nevertheless, the consumption of natural supplements needs to be balanced to avoid negative effects related to overuse or interaction with other medical treatments [7]. Therefore, risk/benefit evaluations are urgently needed when used in foods or health-promoting formulations in order to avoid negative impacts [4].

## 3. Bioactive Compounds and Chemical Composition of *L. barbarum* L.

The bioactive composition of plants is influenced by many factors, such as variety, ripeness, geographic location, and climatic conditions. Since during fruit growth, physiological, biochemical, and molecular changes occur, ripeness might be the most influential factor in the fruit’s bioactive composition [31,32]. Therefore, deep knowledge about the ripening stage of goji berries is needed to determine the best stage and obtain the most adequate bioactive compounds. Yet, in general, various researchers have reported that goji berries have a remarkable concentration of antioxidants, fat, dietary fibers, essential amino acids, valuable trace minerals, and vitamins [3,6,13,23,30,33,34]. In the next subsections, the different classes of bioactive compounds present in goji berries will be deeply analyzed.

### 3.1. Phenolic Compounds

Phenolic compounds act as a defense mechanism to provide adaptation and survival capacity in adverse environmental conditions to plants, such as protection against ultraviolet radiation (UV), pathogen aggression, parasites, and predators [5,35]. These compounds usually add nutritional and functional value, contributing to the fruit’s organoleptic characteristics, such as astringency, bitterness, and aroma. Simultaneously, they guarantee outstanding biological activities and pro-healthy properties against oxidative stress [5,13,31], being capable of delaying, preventing, and inhibiting oxidation by scavenging free radicals and, therefore, reducing oxidative stress [35].

Reactive oxygen species (ROS) naturally occur in living organisms, being involved in processes such as proliferation and apoptosis [25]. However, when the quantities of ROS, such as superoxide radicals (O_2_^·−^), hydrogen peroxide (H_2_O_2_), and hydroxyl radicals (OH^·-−^), overcome the activity of endogenous antioxidant mechanisms, namely antioxidant enzymes (e.g., superoxide dismutase (SOD), catalase (CAT), glutathione peroxidase (GPx)) and non-enzymatic molecules (e.g., glutathione (GSH), ascorbic acid, α-tocopherol), a state of oxidative stress is initiated [10,25,35].

Oxidative stress is implicated in the aging process as well as in several pathologies (e.g., cardiovascular dysfunction, various typologies of cancer, inflammation, rheumatism, diabetes, rheumatoid arthritis, pulmonary emphysema, dermatitis, cataract, neurodegenerative diseases, endothelial cell dysfunction, and several autoimmune diseases linked to degenerative processes of aging) that frequently lead to invalidity or death [3,9,13]. When the endogenous antioxidant mechanisms are not enough to stop and prevent oxidative stress, supplementation is needed to strengthen the antioxidant state and the cell defense mechanisms of organisms [25]. Fruit phenolic compounds are excellent candidates to perform this scavenging effect against ROS and other radical species [35].

Phenolics, flavonoids, and carotenoids are intrinsically related to nutritional and health promotion capacity; therefore, their quantification may help to clarify some activities of natural products [36]. Table 1 summarizes the total phenolic compounds (TPC), total flavonoid content and total carotenoid content (TCC) of goji berries, according to different authors.

As can be observed in Table 1, the values achieved for the different assays (TPC, TFC, and TCC) are slightly different between studies. For example, the TPC varied between 31.6 mg GAE/100 g dw [2] and 3000 mg GAE/100 g dw [12]. These variations are mostly due to the different postharvest techniques used that mainly affect the bioactive components present, as well as the extraction solvents employed and the samples’ geographical origin. Islam et al. [2] studied the phenolic profile, antioxidant capacity, and carotenoid content of dried goji berries harvested in China and extracted with a mixture of acetone/water/acetic acid (70:29.5:0.5). According to the authors, the TPC achieved a value of 31.6 mg GAE/100 g dw, while the TFC was 28.3 mg CAE/100 g dw. These values were considerably lower than the ones reported by Magalhães et al. [12]. Despite the same geographical origin, the authors lyophilized the samples and extracted them for 5 days using methanol (80%), which could justify the huge differences observed.

A study conducted by Donno et al. [13] using goji berries supplied by a farm located in Alzate di Momo, Northern Italy, reported the presence of organic acids (4461.02 mg/100 g fw) and polyphenolic compounds (12,697.90 mg/100 g fw). Meanwhile, in a study [31] using goji berries provided by a planting base in Gansu Province, China, the researchers identified nine phenolic compounds by UPLC-MS/MS, including quercetin, isoquercitrin, chlorogenic acid, ferulic acid, *p*-coumaric acid, caffeic acid, isorhamnetin, cinnamic acid, and rutin, being rutin, isoquercitrin, and chlorogenic acid. Similarly, Pires et al. [30] extracted goji berries supplied by a Portuguese company and reported the presence of nineteen phenolic compounds (71 mg/g dw): eight flavonols (27.6 mg/g dw), seven phenolic acid derivatives (32.7 mg/g dw), one flavan-3-ol (10.4 mg/g dw), and three chlorogenic acids (25.07 mg/g dw). According to the authors, the principal phenolic compounds quantified were quercetin-3-*O*-rutinoside (16.6 mg/g dw) and *p*-coumaric acid (12.3 mg/g dw). Nardi et al. [3] conducted a phytochemical analysis on a methanolic extract of goji berries, revealing the presence of phenolic compounds (142.2 mg/100 g of extract) and flavonoids (74.5 mg/100 g of extract), while the qualitative analysis identified rutin and quercetin as the principal compounds. Wojdyło et al. [36] highlighted the carotenoid content of goji berries from new cultivars in Poland (212.94 mg/100 g dw). The authors identified different types of carotenoids, namely zeaxanthin (84.54 mg/100 g dw), β-carotene (19.35 mg/100 g dw), neoxanthin (16.04 mg/100 g dw) and cryptoxanthin (72.29 mg/100 g dw).

### 3.2. Nutritional Composition

A study carried out by Bora et al. [23] attested to the presence of carbohydrates (46 g/100 g of fw), dietary fibers (16 g/100 g fw), proteins (13 g/100 g fw) and fat (1.5 g/100 g fw) in goji berries. In another study, Ilić et al. [33] reported the presence of moisture (75.32 g/100 g of fw), carbohydrates (16.93 g/100 g fw,), dietary fiber (3.63 g/100 g fw), protein (1.98 g/100 g fw), fat (1.15 g/100 g fw), and ash (0.84 g/100 g fw) in *L. barbarum* berries. Similarly, Pires et al. [30] evaluated the presence of carbohydrates (87 g/100 g dw), proteins (5.3 g/100 g dw), fat (4.1 g/100 g dw), and ash (3.21 g/100 g dw) in dried goji berry fruits and stems. The same authors also reported the presence of soluble sugars (27.9 g/100 g dw), such as fructose (12.7 g/100 g dw), glucose (14.4 g/100 g dw), and sucrose (0.8 g/100 g dw). However, different authors [10,16,20,21,38,39,40,41] attested that polysaccharides are the major carbohydrates present in goji berries, being the principal active ingredients isolated from the fruit. Among them, water-soluble polysaccharides, homogeneous polysaccharides, pectin polysaccharides, acidic heteropolysaccharides, and arabinogalactans (composed of arabinose, glucosamine, galactose, glucose, xylose, mannose, fructose, ribose, galacturonic acid, and glucuronic acid) are the most prevalent.

Regarding organic acids, Pires et al. [30] stated that citric, succinic, and oxalic acids (respectively, 1.29 g/100 g dw, 0.77 g/100 g dw, and 0.010 g/100 g dw) were detected, as well as tocopherols, namely α-tocopherol and δ-tocopherol (0.23 mg/100 g dw and 0.09 mg/100 g dw, respectively). The authors also determined the fatty acids content (4.1 g/100 g dw), more specifically detecting sixteen fatty acids, with polyunsaturated fatty acids being the predominant group, namely linoleic acid (53.4%), oleic acid (16.5%) and palmitic acid (12.77%). In parallel, Ilić et al. [33] reported that the most abundant fatty acids were linoleic (52.1%), oleic (23.6%) and palmitic (17.6%) acids, accounting for 95% of the total fatty acids, which is in concordance with Skenderidis et al. [32], who reported concentrations of 37.89–43.96%, 16.71–20.07%, and 15.08–21.79%, respectively.

Concerning the mineral content, numerous studies showed that the principal minerals present in goji berries are potassium, sodium, and calcium. Bora et al. [23] reported that 100 g of goji berries contain 434 mg of potassium, 60 mg of calcium, 5.4 mg of iron, and 1.5 mg of zinc. Llorent-Martínez et al. [6] reported a higher amount of potassium (1460 mg/100 g), sodium (550 mg/100 g), and calcium (50 mg/100 g), while Ilić et al. [33] quantified potassium (445.12 mg/100 g dw), phosphor (231.52 mg/100 g dw), sodium (74.57 mg/100 g dw), and calcium (29.02 mg/100 g dw).

In what concerns vitamins, ascorbic acid (48.94 mg/100 g fw) and tocopherols (0.33 mg/100 g dw) have been described in goji berries [13,14,30,33,41]. Vitamin E, also known as α-tocopherol, is the major liposoluble antioxidant present in the cells’ antioxidant defense system, being able to inhibit membrane lipidic peroxidation [5,42], while vitamin C, also known as ascorbic acid, is an important antioxidant compound of goji berries [10].

Table 2 summarizes the nutritional composition and phenolic composition of goji berries according to the data reported by different authors.

The variations observed between the different studies may be explained by several reasons. As mentioned earlier, a broad number of biological variables interfere with the bioactive composition of fruits, such as ripeness, geographic origin, or climatic conditions [26,31,32,36]. On the other hand, the extraction techniques employed, the extractor solvents used, and the quantification techniques employed, may lead to different results [9,43]. Generally, mature fruits are associated with huge amounts of bioactive compounds with pro-healthy effects.

## 4. Biological Activities of *L. barbarum* L.

Goji berries have been used for thousands of years as herbal medicines in Asian countries due to their rich nutritional value, medical properties, and biological activities [2,13,23,31]. Several studies highlighted the pro-healthy effects of goji berries, particularly in regarding their antioxidant [2,4,12,13], anti-tumor [4,10,15,40], antimicrobial [10,24], hypoglycemic [10], hypolipidemic [10,14], anti-mutagenic [40], immunomodulatory [16], prebiotic [10,23,24], anti-aging [10], anti-fatigue [10] and neuroprotective activities [12]. These biological activities have been closely related to the fruits’ phenolic composition, particularly phenolic acids, flavonoids, carotenoids, and tannins, which are associated with different biological effects [2,5,23,31,42]. This correlation led to reports of health benefits associated with liver, kidney, eyesight, immune system, circulation, and longevity disorders [4,19,24,30,36]. The following sections will discuss each biological activity in detail.

### 4.1. Antioxidant Activity

Oxidative stress is a phenomenon that occurs due to an imbalance between pro-oxidants and antioxidants, being a consequence of the excessive production of reactive species [3]. In some situations, external antioxidant supplementation is required to reestablish the balance, and fruits’ phenolic compounds are well-known for this capacity [35]. The main contributors to the antioxidant capacity of food, especially fruits and vegetables, are phenolic compounds, particularly phenolic acids and flavonoids, carotenoids, tocopherol, polysaccharides, ascorbic acid, and condensed tannins [2,5,10,36,42]. These molecules stimulate the antioxidant defenses by delaying, inhibiting, or preventing the free radicals from damaging proteins, DNA, and lipids, as well as by scavenging free radicals by hydrogen atom transfer or electron donation or enhancing endogenous antioxidant defenses, such as antioxidant enzymes (SOD, CAT, GPx, …) [3,10,13,35].

Different assays are usually employed to evaluate the radical and antioxidant scavenging capacity. The most frequently applied include the 2,2-diphenyl-1-picrylhydrazyl radical (DPPH^•^), the 2,20-azino-bis(3-ethylbenzothiazoline-6-sulphonic acid) radical cation (ABTS^•+^), scavenging activity, and ferric reducing antioxidant power (FRAP) [10,43]. Table 3 summarizes the reported data on the antioxidant and antiradical activities of goji berries.

A study conducted by Islam et al. [2] assessed the TPC (3.16 mg GAE/g dw), TFC (2.83 mg CAE/g dw), condensed tannin content (CTC; 1.08 mg CAE/g dw), and monomeric anthocyanin content (MAC; 0.24 mg MAC/g dw) of goji berries, along with DPPH (16.65 µmol TE/g dw), ABTS (59.14 µmol TE/g dw), and FRAP (3516.75 mmol Fe^2+^E/g dw) capacity. The results showed a positive linear correlation between DPPH, ABTS, FRAP, and phenolic compounds (0.786, 0.643 and 0.856, respectively), flavonoids (0.857, 0.714 and 0.786, respectively), condensed tannin (0.429, 0.714 and 0.643, respectively) and anthocyanin content (0.643, 0.786 and 0.857, respectively), supporting the idea that phenolic compounds are the main contributors to the antioxidant/antiradical activities of goji berries, which is in line with previous studies [35]. Another study [13] evaluated the TPC (268.5 mg GAE/100 g fw), FRAP (19.36 µmol Fe^2+^E/g fw), and total bioactive compound content (TBCC; 5806.80 mg/100 g fw) of a methanolic extract of goji berries and identified and quantified the principal bioactive compounds present, namely polyphenols (12,697.90 mg/100 g fw) and organic acids (4461.01 mg/100 g fw). Afterwards, the authors verified the correlation between the antioxidant activity and the variables evaluated, reporting a strong positive correlation between the antioxidant capacity and phenols (0.8290), organic acids (0.8606), TPC (0.9996) and TBCC (0.8363), which attests to the antioxidant capacity of goji berries.

Similarly, after identifying and quantifying the polyphenols (97.23 mg/100 g dw), carotenoids (212.94 mg/100 g dw), organic acids (24.7%), and flavonols (75.3%), Wojdyło et al. [36] determined the antioxidant/antiradical activities of goji berries by FRAP (1.44–6.30 mmol TE/100 g fw) and ABTS assays (1.60–6.83 mmol TE/100 g fw). The Pearson correlation allowed the verification of a linear correlation between ABTS or FRAP and the total polyphenolic compounds (0.523 and 0.038, respectively), phenolic acids (0.277 and 0.328, respectively), and flavonols (0.531 and 0.409, respectively). However, goji berries have a high content of carotenoids; therefore, the Pearson correlation between the ABTS or FRAP and the total carotenoids (0.462 and 0.409, respectively), zeaxanthin (0.381 and 0.315, respectively), β-carotene (0.316 and 0.277, respectively), neoxanthin (0.546 and 0.527, respectively) and cryptoxanthin (0.411 and 0.379, respectively) proved the importance of these compounds for the antioxidant power of this fruit.

Meanwhile, Pehlİvan Karakaçs et al. [20] evaluated the levels of antioxidant enzymes (namely SOD, CAT, and GPx) and malondialdehyde (MDA), a characteristic molecule of the oxidative state, when a *L. barbarum* polysaccharide (LBP) extract was orally administrated to rats for 4 weeks. The LBP extract was prepared by crushing 200 g of dried fruits and performing 2 extractions with 600 mL of a chloroform:methanol (2:1) solution at 80 °C. The results showed that the SOD, CAT, and GPx levels increased, while the MDA levels decreased in the blood serum of rats treated with LBP extract, supporting the in vivo antioxidant power of goji berries.

Skenderidis et al. [25] also demonstrated the antioxidant activity of an aqueous extract of *L. barbarum* berries cultivated in Greece and extracted by ultrasound-assisted extraction (UAE). The results of the XTT assay performed in C2C12 muscle cells showed cytotoxicity in concentrations higher than 125 µg/mL. The levels of GSH were also evaluated by flow cytometry after exposing C2C12 muscle cells to the extract (25 µg/mL and 100 µg/mL), and the results revealed an increase of 127.5% and 189.5%, respectively, when compared to the control. As for the thiobarbituric acid reactive substance (TBARS) levels, a marker of lipid peroxidation, the decreases of 21.8% and 9.4% after exposure to 25 µg/mL and 100 µg/mL, respectively, attested to the antioxidant capacity of goji berries extracts.

### 4.2. Anticancer Activity

Despite the extensive research and the advances in cancer treatments made in recent years, cancer is still a worldwide problem [17,40]. The knowledge that several polyphenol-rich extracts from natural matrices have been suggested as promising anticancer agents with few side effects, being associated with lower risks of cancer and cancer mortality, has increased consumers’ interest in fruits and natural matrices [5,6,35].

Kwaśnik et al. [17] performed a 3-(4,5-dimethylthiazol-2-yl)-2,5-diphenyltetrazolium bromide (MTT) assay with human natural killer cells (NK-92) against human colon cancer cell line LS180 after 48 h of incubation in the presence or absence of an ethanolic extract of goji berries in different concentrations (1–500 µg/mL). According to the authors, in the absence of the extract, the strongest anticancer effect obtained (with the elimination of 91% of the cancer cells) occurs when LS180 and NK-92 were used in a ratio of 1:1. In the presence of extract, using the same ratio, 94.8% of the LS180 cells were eliminated. Therefore, goji berries extract reduced by 5%, 12.8% and 20.6%, respectively, the proliferation of LS180 using concentrations of 1, 2.5 and 5 μg/mL. When LS180 and NK-92 cells, in a ratio of 2:3, are exposed to goji berries extract in concentrations of 2.5 and 5 μg/mL, the viability of LS180 cells is reduced to 96.5% and 98.1%, respectively. These results support the chemopreventive properties of goji berry extract, depending on the dose and number of lymphocytes used. Probably, this is due to the immunomodulatory properties of goji berries, which increase the viability and proliferation of NK cells and promote their recognition and elimination capacity.

Another study assessed the proliferation and apoptotic and necrotic effects of different concentrations of an ethanolic extract of goji berries in the T47D human breast cancer cell line [44]. The MTT assay results attested to a strong decrease in cell proliferation after exposure to the highest extract concentration (1 mg/mL) (70%, 55.7%, and 51.4% after 24, 48, and 96 h, respectively). The bromodeoxyuridine (BrdU) cell proliferation assay supported these results, showing a sharp decrease in the proliferation of T47D cells, similar to the Neutral Red (NR) cell viability assay, which demonstrated a slight decrease in the T47D cell viability. The Western blotting analyses employed to evaluate the expression of p21 and p53 proteins, cyclin-dependent kinase 6 (CDK6), and cyclin D1 highlighted the significant increase in p21 and p53 protein expression as well as a slight decrease in CDK6 and cyclin D1 expression. Regarding T47D cells, an increase in the apoptotic percentage was detected (37%, 61.6%, and 88% when treated with 0.1, 0.5, and 1 mg/mL of extract, respectively), as well as a significant necrotic change at 0.5 mg/mL of extract for propidium iodide and Hoechst solution staining. These results attested that the anticancer activity of goji berries extract is due to apoptotic effects through the mitochondrial pathway. To confirm these results using Western blotting, the authors demonstrated a dose-dependent significant increase in pro-apoptotic Bax protein expression and a decrease in anti-apoptotic BclxL protein expression after treatment of T47D cells with the extract for 48 h when compared to the control.

A study using hepatoma cells (SMMC-7721 and HepG2), cervical cancer cells (HeLa), gastric carcinoma cells (SGC-7901), and human breast cancer cells (MCF-7) determined the influence of *L. barbarum* fruits’ crude polysaccharides on the inhibition of cancer cell growth via cell arrest and apoptosis [40]. According to the authors, the fruits’ crude polysaccharides were obtained by water extraction, alcohol precipitation and deproteinization, and fractional precipitation with gradient concentrations of ethanol (30%, 50%, and 70%), originating different fractions (LBGP-I-1, LBGP-I-2, and LBGP-I-3). Through an MTT assay, all *L. barbarum* fruit polysaccharide fractions showed a remarkable inhibition of SMMC-7721, HeLa, and MCF-7 cell growth in a dose-dependent manner, with MCF-7 cells being more sensitive to the LBGP-I-3 fraction (with a cell viability reduction to 48.96%). To study the inhibitory mechanism behind this result, the cell cycle of MCF-7 cells treated with LBGP-I-3 at 1000 µg/mL was analyzed by flow cytometry. The results showed a percentage of 72.76%, 24.21%, and 3.04% of cells in the G0/G1, S, and G2/M phase, respectively, suggesting that LBGP-I-3 arrested the MCF-7 cell cycle at the G0/G1 phase. AO-EB and DAPI staining were used to detect basic morphological changes in apoptotic cells, with the results attesting that the LBGP-I-3 fraction induces the MCF-7 apoptosis. The results also supported that the apoptotic effect was caused by the increased expression of pro-apoptotic Caspase-3, 8, and 9 proteins as well as the decrease in the Bcl-2/Bax ratio and mitochondrial membrane potential. A significant decrease in T-SOD and CAT activities, as well as GSH-Px activity and GSH content, coupled with the enhancement of the MAPK signaling pathway, down-regulating p-ErK1/2 levels and up-regulating p-JKN and p-p38 levels, was also observed.

### 4.3. Antimicrobial Activity

The phytochemicals present in plants, particularly phenolic acids, flavonoids, and tannins, exert their antimicrobial effects by complexing with extracellular and soluble proteins, leading to the disruption of the microbial membrane, metal ion deprivation, and interactions with enzymes [30,45]. Another way of explaining the antimicrobial activity of polyphenols is their structural features, as well as the pH and sodium chloride concentration, which results in physiological changes in the microorganisms and, eventually, cell death [45].

A study carried out by Kabir et al. [45] confirmed that chlorogenic acid, one of the principal phenolic acids detected in goji berries, exhibited a bacteriostatic and bactericidal effect against *Escherichia coli*. The bacteriostatic effect was assessed by measuring the optical density by determining the growth inhibition of chlorogenic acid when added to a culture of *E. coli*. Regarding the bactericidal effect, it was assessed by using mid-logarithmic phase cell cultures (10^6^ cells/mL) at different doses (2.5, 5.0, and 10 mM), treatment times (0, 1, 3, and 6 h), temperatures (20, 37, 45, and 50 ⁰C), and pH conditions (8.0, 7.0, 6.0, 5.0, and 4.0). The overall results demonstrated a synergetic antimicrobial effect expressed by chlorogenic acid and related compounds and a dose, temperature, and time-dependent bactericidal effect. The authors stated that the bactericidal effect shown by chlorogenic acid may be due to the capacity to promote physiological changes on the microbial cell membrane that result in cell death. This study corroborated the results achieved by Ilić et al. [33], which evaluated the antimicrobial activity of goji berries from Serbia. According to the authors, the methanolic extract of goji berries presented mild antimicrobial activity against Gram-positive and Gram-negative bacteria as well as yeast, with remarkable activity against *Klebsiella pneumoniae*, *Salmonella abony,* and *Pseudomonas aeruginosa.* In another study, Mocan et al. [46] also explored the antimicrobial activity of *L. barbarum* flowers. According to the authors, the antimicrobial activity was mild against Gram-positive (namely *Staphylococcus aureus*, *Bacillus subtilis,* and *Listeria monocytogenes*) and Gram-negative (*Salmonella typhimurium*) bacteria and lacking against *E. coli*, oppositely to what was observed for goji berries.

### 4.4. Anti-Inflammatory Activity

The inflammatory response is initiated by the stimulation of innate immunity, the release of immune effectors, the inhibition of enzymes, and the production of pro-inflammatory mediators, such as tumor necrosis factor-α (TNF-α), interleukins (IL), and transcription factor nuclear factor kappa B (NF-kB) [3]. If over-expressed, inflammation can cause numerous complications in patients, being an important hallmark of diseases or pathological conditions, such as cancer, neurodegenerative diseases, respiratory pathologies, and several autoimmune diseases [3,5]. Natural extracts, typically rich in alkaloids, flavonoids, terpenoids and tannins, have been associated with anti-inflammatory effects [5]. A study conducted by Nardi et al. [3] addressed the anti-inflammatory effect of *L. barbarum* berries using paw edema carrageenan, an acute model of inflammation widely employed to evaluate the anti-inflammatory effect of natural products. Briefly, the right paw of mice was injected with 450 µg of carrageenan, and the left paw was injected with the same volume of sterile phosphate-buffered saline (PBS) (control). After established conditions, the animals were orally administered with a methanolic extract of goji berries in doses of 50 mg/kg and 200 mg/kg, 12 h after 12 h, for 10 days. The authors stated that after the carrageenan injection, neutrophils migrate to the inflamed paw and release enzymes, such as myeloperoxidase, which increase ROS production and induce an inflammatory state. The results attested to the anti-inflammatory capacity of the compounds present in goji berries, with the doses of 50 mg/kg and 200 mg/kg leading to a reduction of paw edema in 38% and 63.8% of mice, respectively.

### 4.5. Immunomodulatory Activity

Immune response suppression is usually associated with the development of immunological diseases. The actual research is focused on finding immunomodulating agents capable of preventing and treating these disorders [47]. Goji berries have been reported as a support to the immune system [6,47], either by regulating the expression of immune factors, such as cytokines and cell adhesion molecules [16,39], or by promoting the proliferation and activity of immune cells, such as natural killer cells [17] and lymphocytes [47].

Different authors [16,24,39,47] stated that the immunomodulatory effects of *L. barbarum* fruits are greatly related to LBP, one of the major active ingredients isolated from berries. LBP is a mixture of proteoglycans and polysaccharides that mainly consists of arabinose, galactose, glucose, xylose, and a small amount of rhamnose, mannose, and galacturonic acid as its glycosidic part. It was reported that LBP is an adjuvant that improves the immune responses against vaccines and increases humoral immunity [39], probably due to an increased expression of IL-2 and TNF-α, molecules involved in immunomodulation [16]. To assess the immune function alteration during the administration of LBP, Zhu et al. [39] divided fourteen mice into two groups and administered a dose of 0.1 mL/10 g body weight of LBP powder for 14 days in the first group, the experimental group, and the same volume of physiological saline via intragastric administration in the second group, the control group. After the experimental period, blood samples were collected, and the animals were sacrificed to excise the spleen and thymus. The viscera indices were measured (thymus or spleen index = weight of thymus or spleen (mg)/body weight (1000 mg)), and the concentrations of immune factors (namely, transforming growth factor-β (TGF-β), interferon-γ (IFN-γ), and IL-6) in the serum were detected using a commercial enzyme-linked immunosorbent assay (ELISA) kit. The results supported the theory that LBP can enhance the innate immune response since the spleen and thymus index of mice from the experimental group (5.12 mg/1000 mg and 4.12 mg/1000 mg, respectively) were significantly higher (*p* < 0.05) when compared to the control group (namely 4.19 mg/1000 mg and 2.86 mg/1000 mg). Moreover, the concentration of the immune factors TGF-β and IL-6 in the experimental group (namely, > 500 pg/mL and ≈ 100 pg/mL) were also significantly higher (*p* < 0.05) when compared to the control group.

It has also been reported that phenolic amides from the nonpolysaccharide fraction of *L. barbarum* fruits can not only in vitro modulate the proliferation of B and T cells but also enhance the immune cell factors (IFN-γ, IL-2, and IL-10) [47]. Prednisone-induced immunodeficient mice were intragastrically administered with a phenolic amid extract, showing a significant spleen cell proliferation (*p* < 0.01) when compared to the prednisone-induced immunodeficient mice intragastrically administered with an ethanolic extract of goji berries or an LBP extract. The immunological cytokines (IFN-γ, IL-2, and IL-10) were also significantly enhanced (*p* < 0.01) in mice treated with the ethanolic goji berries extract, LBP extract, and phenolic amide extract when compared to the control. Overall, the total phenolic amides had the strongest immunity-activating effects.

Since natural killer cells are one of the first elements from the innate immune system to act, Kwaśnik et al. [17] studied the cell viability and proliferation of human natural killer cells (NK-92) in the presence of an ethanolic goji berry extract. The authors highlighted that goji berry extract has the capacity to enhance NK cell proliferation by 61.0% in concentrations ranging between 1 and 250 µg/mL.

### 4.6. Prebiotic Activity

Prebiotics are non-digestible ingredients that can selectively promote the growth and activity of specific bacteria, modulating the gut microbiota [39]. Goji berries have demonstrated a gut microbiota positive modulating effect [18,39], probably due to phenolic compounds or polysaccharides present, such as LBP [43]. The prebiotic activity of goji berries was attested to by Skenderidis et al. [18], who cultivated strains of *Bifidobacterium* and *Lactobacillus* in the presence and absence of different encapsulated goji berry extracts. According to the authors, the extract with higher amounts of polyphenols and polysaccharides stimulated the growth and proliferation of probiotic strains on a larger scale, mostly *Bifidobacterium* strains, when the cultures were submitted to a gastrointestinal environment (simulated gastric and intestinal juices). Another study [39] assessed the prebiotic activity of LBP in vitro through the growth of *L. acidophilus* and *B. longum*. Both strains were properly cultivated, and different concentrations of LBP powder (experimental group) and glucose (control group) were administered. These experiments highlighted the positive effect of LBP on the growth of both strains when compared to the control group. The authors also evaluated the effects of LBP intake on the composition of cecal gut microbiota [39]. After mice were administered a dose of 0.1 mL/10 g body weight of LBP powder for 14 days, the cecal content samples were collected, and the microbial DNA was extracted and amplified. The results showed a clear modification of the gut microbiota after treatment, with the dominant bacterial communities being *Firmicutes* and *Proteobacteria.* Furthermore, the percentage of beneficial bacteria, such as *Akkermansia*, *Lactobacillus* and *Prevotellaceae*, significantly increased when compared to the control.

### 4.7. Neuroprotective Activity

Since neurological damage may occur as a consequence of oxidative stress and inflammation, goji berries have been associated with neuroprotective effects [43]. A study conducted by Fernando et al. [19] assessed the neuroprotective activity of a goji berry powder (GBP) against the development of Alzheimer’s disease (AD). The authors observed that diets rich in antioxidants can directly affect amyloid beta levels and influence the development of AD. To test this theory, human neuroblastoma BE(2)-M17 cells were treated with and without 20 µM of amyloid beta 42 and exposed to different concentrations of GBP (0.6, 0.9, 1.2, 1.5 and 1.8 µg/mL). The GBP significantly increased the cell viability up to 105% (GBP at 1.2 µg/mL), while the Western blot analysis showed a significant reduction in the amyloid beta up to 20% (GBP at 1.5 µg/mL). Furthermore, the ELISA attested to a 17% reduction of the amyloid beta in amyloid beta-induced neuronal cells when compared to the control (GBP at 1.5 µg/mL). Other authors [16,24] also stated that the major active ingredient isolated from goji berries, LBP, has ocular and neuroprotective effects [20]. In another study, Pehlİvan Karakaçs et al. [20] reported that LBP has a neuroprotection effect in female rats by performing behavioral tests and immunohistochemistry analyses. The animals were submitted to ovariectomy and daily treated with oral low and high doses of polysaccharides obtained from *L. barbarum* fruits (20 and 200 mg/kg) for 4 weeks. According to the behavioral test performed, the LBP present in goji berries can decrease stress-induced anxious behavior in rats. The behavioral disruption could be caused by the accumulation of ROS in the brain, which increases oxidative stress, leading to mitochondrial dysfunction in neuronal cells and apoptosis. However, behavioral disruption can also be caused by variations in neurotransmitter levels in some brain regions, such as the hippocampus. The neuroprotective effect of LBP was demonstrated by the increased levels of brain-derived neurotrophic factor, which promotes neuronal proliferation, survival, and maintenance of neuron structure and function in the hippocampus region.

### 4.8. Antihyperglycemic Activity

Diabetes is a complex chronic disease caused by metabolic disorders of carbohydrates, lipids, lipoproteins, and increased oxidative stress [43]. It is characterized by the inability to maintain blood glucose levels within healthy ranges, leading to hyperglycemia [36]. Drug treatment is inevitable; however, it is often expensive or unavailable [36]. In these cases, a change in patient lifestyle and eating habits is extremely important [21,38,43]. Therefore, industries are encouraged to find alternative compounds with antihyperglycemic properties, mostly from natural sources, such as goji berries [5,10]. Enzyme inhibitors are good candidates for the treatment of non-insulin-dependent diabetic patients [36]. Since pancreatic α-amylase and intestinal α-glucosidase are enzymes responsible for the hydrolysis of carbohydrates into monosaccharides, such as glucose, the capacity of goji berries to inhibit these enzymes proves the antihyperglycemic effect of this fruit [36]. According to Wojdyło et al. [36], the inhibition of α-amylase and α-glucosidase was, on average, 63% and 10%, respectively.

Most of the experiments that report the antihyperglycemic activity of goji berries have attributed this property to LBP [21,25,38]. Cai et al. [21] performed a clinical trial to study the antidiabetic efficacy of LBP. Sixty-seven patients diagnosed with type II diabetes were divided into two groups and treated twice daily, for three consecutive months, with capsules of 300 mg of microcrystalline cellulose in the control group and capsules with 150 mg of LBP power and 150 mg microcrystalline cellulose in the experimental group. The results of the oral metabolic tolerance test reported a significant decrease in serum glucose levels when compared to the control (−7.86% vs. 1.61%), as well as serum insulin (−56.71% vs. −8.73%). Regarding the insulinogenic index (insulin/glucose), the results attested to a noticeable increase (−0.98% vs. 0.04%, respectively, for the experimental and control groups). During the experimental period, the patients were asked not to change their previous therapies in order to compare the antidiabetic effect of LBP with or without the hypoglycemic medicines. After 3 months of treatment, the patients without hypoglycemic drugs showed a significant decrease in serum glucose, serum insulin, and the homeostasis model assessment index of insulin resistance, while the patients with hypoglycemic drugs did not present significant alterations, demonstrating that LBP has profound hypoglycemic efficacy when disassociated from hypoglycemic drugs.

The study developed by Zhao et al. [38] focused on the effect of LBP extract on diabetic rabbits (induced by Alloxan), evaluating the extract’s effect on diabetic nephropathy (DN). The experimental period lasted for 12 weeks, in which twenty-five rabbits were divided into five groups: group (I)—control group; group (II)—DN control group; group (III)—LBP prevention group from developing DN; group (IV)—positive DN control group using Telmisartan drug (10 mg/kg/day in 3 mL); group (V)—LBP treatment group using the LBP (10 mg/kg/day in 3 mL) as DN treatment. The results demonstrated that LBP not only helps after the development of DN but also benefits the prevention of complications since group (V) showed improvements in fasting glucose tolerance when compared to group (II), while group (III) had better results than group (V). This study also highlighted that LBP can be more efficient in the treatment of DN than standard drugs (in group (V), the kidney weight index (KWI) was significantly higher (*p* < 0.01) than in group (IV)). As for kidney function, even though LBP may be able to protect this organ from injuries, it cannot entirely treat it since the serum urea nitrogen and creatinine levels from groups (III), (IV) and (V) were significantly lower (*p* < 0.01) that in group (II). No significant changes were observed regarding group (I).

### 4.9. Antihyperlipidemic Activity

In addition to the biological activities described above, the antihyperlipidemic effect of goji berries has also been reported. Pai et al. [14] fed murine models with a high-fat diet (5 g of deoxycholic acid and 300 g of warm coconut oil mixed with 700 g of powdered rat chow per day) for 45 days to induce a state of hyperlipidemia. In the last 30 days of the study, a group of animals was orally treated daily with 10 mg/kg and 20 mg/kg of a 50% hydroalcoholic extract of *L. barbarum* fruits. The results showed a positive antihyperlipidemic activity, with the extract promoting a significant decrease in the triglyceride levels for both doses as well as a significant decrease in very low-density lipoprotein cholesterol at the highest dose, a significant increase in the high-density lipoprotein cholesterol at the lowest dose, and a dose-dependent decrease in the total cholesterol and low-density lipoprotein cholesterol levels. Additionally, the results obtained with the animals treated with the *L. barbarum* fruits and the animals treated with the standard antihyperlipidemic drug atorvastatin were similar, reinforcing the positive antihyperlipidemic activity of goji berries. The authors explained that this effect may be caused by more than one compound present in goji berries, namely polysaccharides and vitamins, such as vitamin C. Additionally, riboflavin, ascorbic acid, and coumarin and their synergistic effects may contribute to the hypolipidemic effect observed.

## 5. Potential Applications of *L. barbarum* L.

Goji berries can be used in different industries, such as the food, nutraceutical, or cosmetic industries. Regarding the food industry, it has been reported that the aqueous extracts of goji berries can be employed as potentially prebiotic food additives [10,18].

Goji extract has also been successfully used in meat products to improve their sensory properties and oxidative stability during storage [48]. The addition of 1.0% of goji berry extract and 1.0% of buckwheat flour improved the oxidative stability and quality of modified horse-meat products, resulting in a better smell, taste, and surface color after 21 days of storage [49]. Another study determined the effect of the addition of goji berries or goji berry extract on sausages [48]. The addition of 1% of goji berry extracts effectively suppressed lipolysis and protein/lipid oxidation, reducing the microbial count during storage and preserving the sausages’ color, aroma, and taste. Antonini et al. [50] also evaluated the addition of goji berries to meet formulas and determined that the addition of chia seeds and *L. barbarum* puree (2.5% and 5%, respectively) to beef burgers promotes a higher TPC, an increase of up to 70% of the total antiradical capacity (through the oxygen radical absorbance capacity (ORAC), ABTS, and DPPH) and a decrease in the lipid peroxidation of up to 50%.

The confectionery and bakery industries are also using goji berries and their compounds to improve the functional, sensory, and texture properties of various products [10]. Muffins and cookies enriched with different amounts of goji berry powder or by-products showed an increase in the TPC, insoluble, and soluble fiber contents as well as good sensory properties [23].

Nutraceuticals are foods, beverages, or supplements with high concentrations of bioactive compounds with outstanding health-promoting effects on the human body, being incorporated into consumers’ daily diets [9]. Due to the bioactive compounds present in their fruits, goji berries are being investigated as a potential nutraceutical ingredient [2,3,5]. As previously mentioned, goji berries have been used in herbal medicine for thousands of years [24], along with hundreds of plants cultivated worldwide for their substances to be used in medicine and pharmaceutical formulations [51]. Based on the long-term traditional use of goji berries, this fruit is now generally recognized as non-toxic [10]. However, adverse effects can occur and, depending on the goji berries’ use, a public health risk can be considered [51]. The presence of tropane alkaloids, chemical contaminants, such as pesticides and toxic elements, or some proteins that can cause allergic reactions in sensitive consumers are clearly risks [10]. Therefore, caution and professional guidance should be exercised regarding the safe consumption of natural matrices, such as goji berries, to avoid adverse effects, toxicity, and allergies [52].

Furthermore, the application of this fruit and its components at high levels in different industries, such as the food, pharmaceutical, nutraceutical, or cosmetic industry, can substantially deteriorate the sensory, textural, and overall quality of the final products [23]. More studies need to be conducted to determine to what point the use of this fruit is beneficial for consumers [4]. However, overall, considering the excellent nutritional profile and the positive health effects of goji berries, this fruit can be classified as a “superfruit” [12,13].

## 6. Valorization Prospects of *L. barbarum* L.

During the production and processing of food products, a huge amount of waste is generated, in which a significant amount of bioactive compounds may be present [11]. In the European Union (EU), approximately 88 million tons of waste are generated annually, with associated costs estimated at EUR 143 billion [42]. The last data from the Food and Agriculture Organization of the United Nations (FAO) state that fruit and vegetable processing wastes, such as peels, pomace, flowers, stems, leaves, seeds, and pulps, are the 5th highest contributor (8% of total food waste) [42,53].

In the processing of goji berries, significant amounts of wastes are generated (approximately 10 kg of waste per 90 kg of goji beverage) [23]. In addition to the economic costs associated with these wastes’ disposal, a serious negative impact on the environment is estimated [23]. As a result, industries face the urgent and necessary challenge of implementing greener, more efficient, sustainable, and eco-friendly processing protocols [5]. Regarding processing protocols, since extraction is a critical step in the isolation and purification of bioactive compounds, industries have focused on overcoming the limitations of conventional extraction methods, such as reduced extraction efficiency, high energy employment, and the use of high amounts of solvent wastes [32]. For this reason, new green extraction technologies have emerged [41], such as hot water extraction (HWE), subcritical water extraction (SWE), supercritical fluids extraction (SFE), ultrasound-assisted extraction (UAE) and microwave-assisted extraction (MAE).

Therefore, the valorization of agro-residues and industrial wastes has proved to be an advantage [23,42]. As previously referred, goji berries are ingested in many forms and bring a panoply of beneficial pro-healthy effects to consumers, from nutritional to medicinal and curative approaches. However, their by-products, including leaves, stems, young shoots, and root bark, can also be consumed as part of a traditional diet, and used for medicinal purposes [33]. For instance, a study developed in Portugal [30] compared the composition of goji stems and berries and reported similar values of phenolic compounds (71.9 vs. 71 mg/g dw), organic acids (2.08 vs. 2.07 g/100 g dw), energy (383 vs. 408 kcal/100 g dw), total carbohydrates (78.1 vs. 87 g/100 g dw) and fat (4.6 vs. 4.1 g/100 g dw). In what concerns tocopherols (3.59 vs. 0.33 mg/100 g dw), proteins (7.4 vs. 5.3 g/100 g dw) and saturated fatty acids (68 vs. 26.1%), the stems revealed a high content. However, stems also had higher antioxidant and antiradical capacities (DPPH scavenging activity (EC_50_ = 0.28 mg/mL), reducing power (EC_50_ = 0.23 mg/mL), β-carotene bleaching inhibition (EC_50_ = 0.26 mg/mL), and TBARS inhibition (EC_50_ = 0.07 mg/mL), as well as antibacterial activity against Gram-negative (*Escherichia coli*, *Morganella morganii*, *Pseudomonas aeruginosa*, *Acinetobacter baumannii*) and Gram-positive bacteria (*Staphylococcus aureus*, *Listeria monocytogenes*, *Enterococcus faecalis*) when compared to goji berries [30].

Another study determined the chemical composition (TPC, TFC, and HPLC/MS analysis) and evaluated the antioxidant activity (Trolox equivalent antioxidant capacity (TEAC), hemoglobin/ascorbate peroxidase activity inhibition (HAPX) assay, inhibition of lipid peroxidation catalyzed by cytochrome c and electron paramagnetic resonance (EPR) spectroscopy) of an ethanolic extract of *L. barbarum* flowers [46]. The results show its richness in chlorogenic, *p*-coumaric, and ferulic acids, isoquercitrin, rutin, and quercitrin (similar to what was stated in studies concerning goji berries [3,30,31]) and a TPC and TFC very similar to *L. barbarum* fruits (3.75 and 0.61 mg/g dw vs. 1.45 and 0.75 mg/g dw [3], respectively). Regarding the antioxidant capacity, the results attested to their activity, which was positively correlated with their chemical composition. After an ultrasonic extraction with methanol/water (70:30, *v/v*), an extract of goji berry leaves was evaluated regarding phenolic profile and antioxidant capacity (TEAC and EPR spectroscopy) [54]. The phenolic profile revealed the presence of chlorogenic acid and rutin, as previously reported for goji berries [3,30,31]. Regarding the antioxidant activity, the results showed a high antioxidant power (140 mg TE/g dw and 212 mg FSE/g dw, respectively). Along with the enzyme inhibitory activity (cholinesterase, α-amylase, α-glucosidase and tyrosinase), antimicrobial activity (*S. aureus*, *Bacillus cereus*, *Listeria monocytogenes*, *Enterococcus faecalis*, *Pseudomonas aeruginosa*, *Salmonella typhimurium*, *E. coli*, *Fusobacterium nucleatum,* and *Peptostreptococcus anaerobius*) and antifungal activity (*Aspergillus flavus*, *Aspergillus niger*, *Candida albicans*, *Candida parapsilosis,* and *Penicillium funiculosum*), this study confirms the potential of goji leaves to be used as a valuable source of bioactive compounds. Apart from that, polyphenols from goji berries leaves were also encapsulated in liposomes to improve their delivery and successfully serve as polyphenol carriers, demonstrating a cytoprotective effect on L-929 mouse fibroblast cells [10].

Based on the results detailed above, it can be stated that goji berry by-products, such as fruits skins, pulps, stems, and seeds, have high amounts of dietary fiber, vitamins, minerals, phytochemicals, and antioxidants, being a valuable ingredient for the food industry. Therefore, the incorporation of goji berry by-products, for example, in the bakery industry, may provide benefits to consumers as well as manufacturers, not only due to their nutritional value but also due to the economic advantage that comes from the elimination of the costs required for its disposal [23]. Nevertheless, safety and security studies should be previously performed considering these potential applications.

## 7. Conclusions

Goji berries can be classified as a “superfruit” due to their outstanding phytochemical composition, which includes phenolic acids (up to 32.70 mg/g), flavonoids (up to 38.00 mg/g), organic acids (up to 44.61 mg/g fw), carotenoids (up to 2.13 mg/g dw), carbohydrates (up to 87.00 g/100 g dw), and vitamins (up to 48.94 mg/100 g fw). The main phenolic compounds reported in this fruit are phenolic acids (chlorogenic acid and *p*-coumaric acid) and flavonoids (rutin and quercetin). Aside from these compounds, vitamins (ascorbic acid), carbohydrates (polysaccharides), and carotenoids (zeaxanthin, β-carotene, and cryptoxanthin) are the main compounds responsible for the incredible biological activity observed, such as antioxidant, anti-tumor, antimicrobial, hypoglycemic, hypolipidemic, anti-mutagenic, immunomodulatory, and prebiotic activities. In this regard, goji berries can be used in the food, nutraceutical, and pharmaceutical industries as a source of functional ingredients. Despite this, *L. barbarum* leaves, flowers, and steams are valuable sources of bioactive compounds that can be explored for use in different industries. Even though the valorization of goji by-products helps to reduce the food waste produced during food processing, the high energy required, as well as the use of high amounts of solvents in the extraction processes, are still challenges. In the future, the implementation of green extraction techniques is expected at industrial levels, minimizing the wastes produced and maximizing the extraction of bioactive compounds from goji berries and by-products.

## Figures and Tables

**Table 1:** Total phenolic compounds (TPC), total flavonoid content and total carotenoid content (TCC) of goji berries according to different studies.

TPC	TFC	TCC	Reference
31.6 mg GAE/100 g dw	28.3 mg CAE/100 g dw	23.30 mg CAE/100 g dw	[2]
145.2 mg GAE/100 g dw	74.5 mg QE/100 g dw	-	[3]
1160–1570 mg GAE/100 g dw	-	-	[4]
1413 mg GAE/100 g dw	-	-	[37]
3000 mg GAE/100 g dw	2480 mg QE/100 g dw	-	[12]
268.5 mg GAE/100 g fw	-	-	[13]
449–778 mg GAE/100 g dw	-	400–950 mg/100 g dw	[28]
162.4 mg GAE/100 g fw	214.2 mg HE/100 g fw	41.71 mg/100 g fw	[33]
97.23 mg/100 g dw	-	212.94 mg/100 g dw	[36]

GAE—gallic acid equivalents; CAE—catechin equivalents; HE—hyperoside equivalent; QE—quercetin equivalents; TPC—total phenolic content; TFC—total flavonoid content; TCC—total carotenoid content; fw—fresh weight; dw—dry weight.

**Table 2:** Phytochemical and nutritional composition of *L. barbarum* berries, according to different studies.

Compounds	Amount	Reference
Phenolic compounds Phenolic acids - Chlorogenic acid - *p*-coumaric acid - Ferulic acid - Caffeic acid Flavanols - Rutin - Quercetin Flavan-3-ol - Catechin - Epicatechin	12,697.90 mg/100 g fw 32.70 mg/g - 25.07–1.07 mg/g - 12.30 mg/g - 0.98–0.93 mg/g - 0.93–8.99 mg/g 27.60 mg/g - 16.60–12.84 mg/g - 10.23–9.41 mg/g 10.40 mg/g - 1.34–1.13 mg/g - 2.18–1.98 mg/g	[3,9,13,30]
Organic acids Citric Malic Oxalic Quinic Tartaric	4461.02 mg/100 g fw 254.09 mg/100 g fw 601.43 mg/100 g fw 13.41 mg/100 g fw 2011.73 mg/100 g fw 1580.35 mg/100 g fw	[13]
Carotenoids Zeaxanthin β-Carotene Cryptoxanthin	212.94 mg/100 g dw 84.54 mg/100 g dw 19.35 mg/100 g dw 72,29 mg/100 g dw	[36]
Vitamins Ascorbic acid Tocopherol	2.39–48.94 mg/100 g fw 0.33 mg/100 g dw	[13,34,36] [30]
Carbohydrates Total sugars Soluble sugar - Glucose - Fructose - Sucrose	77.1–87 g/100 g dw 45.60–67.83 g/100 g dw 27.09 g/100 g dw - 14.40–17.32 g/100 g dw - 12.70–21.71 g/100 g dw - 0.80–1.48 g/100 g dw	[30,34] [26,34] [28,30]
Dietary fibers Soluble Insoluble	3.63–16 g/100 g fw 0.90–5.5 g/100 g dw 2.73–11.7 g/100 g dw	[23,33] [30]
Proteins Essential amino acids Non-essential amino acids	5.3–14.3 g/100 g dw 2.139 g/100 g dw 6.728 g/100 g dw	[30,34] [34]
Fatty acids Linoleic acid Oleic acid Palmitic acid	0.39–4.1 g/100 g dw 37.89–53.4% 16.5–23.6% 12.77–21.79%	[30,34]
Ash	0.78–3.21 g/100 g dw	[30,34]
Minerals Potassium Calcium Sodium Iron Phosphor	434–1460 mg/100 g fw 29–60 mg/100 g fw 75–550 mg/100 g fw 5.4 mg/100 g fw 232 mg/100 g fw	[6,23,33]

fw—fresh weight; dw—dry weight.

**Table 3:** Antioxidant and antiradical activities of goji berries according to different authors.

DPPH Assay	ABTS Assay	FRAP Assay	References
4.526 µmol TE/g	129 µmol TE/g	5.324 µmol TE/g	[33]
18.5–13.9 µmol VCE/g	61–54 µmol/g	-	[9]
16.65 µmol TE/g	59.14 µmol TE/g	35.1675 mmol Fe^2+^E/g	[2]
-	16.0–68.3 µmol TE/g	14.4–63.0 µmol TE/g	[36]
8.79–9.35 mg TE/g	24.86–25.12 mg TE/g	16.91–19.52 mg TE/g	[4]
3.12 mg TE/g	-	-	[22]
-	-	42.10 µmol TE/g dw	[28]
-	-	19.36 µmol Fe^2+^E/g	[13]

TE—Trolox equivalent; VCE—vitamin C equivalent; IC_50_—half-maximal inhibitory concentration; Fe^2+^E—Fe^2+^ equivalent; AAE—ascorbic acid equivalent; fw—fresh weight; dw—dry weight.

## Data Availability

Not applicable.
